# Evaluation of the effect of periapical lesions and other odontogenic conditions on maxillary sinus mucosal thickness characteristics and mucosal appearance: A CBCT study

**DOI:** 10.34172/joddd.2021.028

**Published:** 2021-08-25

**Authors:** Selen Ince Yusufoglu, Güzin Neda Hasanoglu Erbasar, Orhan Gülen

**Affiliations:** ^1^Department of Endodontics, Faculty of Dentistry, Ankara Yıldırım Beyazıt University, Ankara, Turkey; ^2^Department of Oral and Maxillofacial Surgery, Faculty of Dentistry, Ankara Yıldırım Beyazıt University, Ankara, Turkey; ^3^Private Practice, Oral and Maxillofacial Radiology, Ankara, Turkey

**Keywords:** Cone-beam computed tomography, Maxillary sinusitis, Periapical periodontitis, Periodontal bone loss, Periapical lesion

## Abstract

**Background.** This retrospective study aimed to investigate the effect of various dental and maxillary sinus variables on maxillary sinus mucosal thickness (MT). The variables included periodontal bone loss (PBL), periapical status, dental restorations of posterior maxillary teeth, and the distance from the root apices to the sinus mucosa.

**Methods.** Cone-beam computed tomography (CBCT) images of the maxillary sinuses (n = 600) in 300 patients were examined. The sinus MT and the distance of the roots from maxillary sinuses were measured. Apical lesions of the roots, PBL, and situations of adjacent teeth were recorded. The relationships between these conditions and MT and characterization of MT were evaluated. The Kruskal–Wallis H test was used to compare groups due to the non-normal distribution of the data. The relationship between categorical variables was analyzed using chi-squared test.

**Results.** There was a significant correlation between maxillary sinus MT and periapical lesions, PBL, and restorations (*P* < 0.05). MT increased as the apical lesions of premolar teeth enlarged (*P* < 0.05, *P* = 0.022). MT increased in cases of mild PBL of molar teeth (*P* = 0.041).

**Conclusion.** In this retrospective study, the MT significantly increased in patients with periapical lesions, inadequate endodontic treatment, increased PBL, and inadequate dental restorations.

## Introduction

The maxillary sinus is a pyramid-shaped air-filled cavity in the body of the maxillary bone. As the maxillary sinus is confined to the nasal cavity and oral cavity, it is more susceptible to pathogenic microorganisms than other paranasal sinuses. Chronic maxillary sinusitis is defined as the inflammation of the sinus membrane covering the paranasal sinus, with signs and symptoms persisting for at least 12 weeks. Chronic maxillary sinusitis might be caused by nasal ostium or oral pathogenic microorganisms.^1^ Odontogenic causes account for 10–12% of cases of maxillary sinusitis.^2^ Odontogenic sinusitis is caused by odontogenic pathologic conditions, including apical inflammatory lesions, endodontic problems, marginal periodontitis, and radicular cysts.^3^ This type of maxillary sinusitis can also occur iatrogenically.^3^ Previous studies have reported that root canal treatment of posterior molar teeth caused odontogenic sinusitis due to the extrusion of root canal filling materials from the apex of associated teeth to the sinus cavity, facilitating the transport of microorganisms from the periapical tissues to the maxillary sinuses.^4,5^

Nonodontogenic sinusitis is the inflammation of the nose and paranasal sinuses. It is identified by nasal congestion or facial pain and reduced olfaction for more than 12 weeks. Odontogenic and nonodontogenic sinusitis have no difference in their clinical symptoms. However, as the microbiology of odontogenic sinusitis differs from that of nonodontogenic sinusitis, the treatment plan in maxillary sinusitis should be tailored to the source of infection.^6^ Although some previous studies focused on the etiology of odontogenic maxillary sinusitis,^7,8^ no studies have investigated the roles of various dental and maxillary sinus variables in the etiology of odontogenic sinusitis. These include dental restorations, the periapical and periodontal status of the teeth, and the distance between the root apex and the base of the sinus mucosa relative to the maxillary sinus mucosa.

Two-dimensional intraoral and panoramic x-rays can be used to assess teeth and odontogenic infections. However, two-dimensional imaging techniques are restricted in assigning periapical and sinus anomalies. Panoramic radiographs can detect mucosal thickness (MT) or fluid deposition but cannot detect sinus anatomy precisely.^9^ To visualize details of the sinus anatomy, a high-precision method is required. Cone-beam computed tomography (CBCT) imaging can provide high-quality three-dimensional images using a low radiation dose at a lower cost than multislice computed tomographic imaging.^10,11^ It enables a comprehensive assessment of the dental root apex and maxillary bone quality and quantity, without superposition and distortion of teeth and surrounding structures.^10,11^

This study aimed to determine the association between the MT of the sinus mucosa and the following variables: sex, the coronal and periapical status of upper premolar and molar teeth, periodontal bone loss (PBL), and distance from the root apex to the sinus mucosa. The hypothesis of this study was that excessive bone loss in the posterior maxillary teeth and periapical lesions (1 mm) would increase the maxillary sinus MT.

## Methods

In this retrospective study, the database was searched to identify patients who had undergone CBCT examinations for various dentomaxillofacial problems in a private CBCT imaging center from December 2018 to June 2019. CBCT images of 530 patients were included in the present study in the first step.

### Inclusion criteria

1. High quality of CBCT images and presence of both maxillary sinuses
2. The presence of at least one of the premolar or molar teeth on the left or right side (completely erupted teeth excluding the third molars)
3. No absence of posterior teeth unilaterally
4. No dental implants
5. No orthodontic appliances
6. No history of any bone disease, no history of facial trauma and oral surgery, no oral pathology according to the anamnesis of the patients

Finally, images obtained from 300 patients with 600 maxillary sinuses (right and left), with mucosal thickening on at least one side, were included.

The CBCT images were evaluated by an endodontist and a maxillofacial radiologist. They examined and calibrated 20 CBCT scans with normal sinus findings or previously diagnosed sinusitis. Each scan was examined in inclined slicing in the axial, coronal, and sagittal planes to evaluate individual teeth/roots.

All the CBCT images were acquired using a Dentri-S model (HDX WILL Corp., Seoul, Korea) CBCT unit operating at 80–100 kVp, 4–10 mA, 0.20-mm voxel size, with an 8–24 seconds acquisition time. Subsequently, the images were reconstructed using specific software (CS 3D imaging, Carestream Health Inc., Rochester NY, USA). Multiplanar reconstructions were obtained and assessed simultaneously by two calibrated evaluators who used a 19-inch LCD monitor (Infoway; Itautec, Taubate, SP, Brazil) under dim light. The density and contrast of the images were adjusted accordingly to assist the evaluators during the identification and measurement of dental pathologies and MT.

### Evaluation of the tooth structure

The tooth structure was classified as follows:

- Healthy: No low-density area in the enamel and dentin layers
- Caries: The presence of a carious lesion limited to the enamel or dentin, which had not progressed to the pulp
- Caries with pulp exposition: The carious lesion reaching the pulp
- Filling: Restored tooth
- Defective filling: Presence of a tooth restoration with a carious lesion
- Root canal treatment: Adequate and proper root canal treatment (firmly filled up to the root apex)
- Inadequate root canal treatment: Non-homogeneous root canal filling and presence of root canal filling 2 mm shorter than the root apex or overflowing from the apex and, with surrounding low-density areas

### Evaluation of periapical status of teeth

A periapical lesion was considered radiolucency in the apical part of the root and the thickness of at least 0.5 mm (twice the normal periodontal ligament width) or > 0.5 mm in sagittal, axial, and coronal measurements. The scoring was made according to the CBCT periapical index, as follows^12^ :a score of 1: 0–0.5 mm; a score of 2: 0.5–1 mm; a score of 3: 1–2 mm; a score of 4: 2–4 mm; a score of 5: 4–8; a score of 6: > 8 mm; cortical expansion: E; cortical destruction: D.

### Evaluation of the periodontal status of teeth

The periodontal status (i.e., PBL) was evaluated as described by Phothikhun et al.^13^ Thus, the periodontal bone was considered normal when the alveolar bone crest was located a maximum of 2 mm from the cementoenamel junction toward the apex. In cases where it was more than 2 mm, measurements were taken from the mesial and distal aspects, and the measurement level was subtracted from the normal level. The percentage of PBL was classified as follows: N = normal periodontal bone height, P1 = mild (< 25% bone loss), P2 = moderate (25–50% bone loss), and P3 = severe (> 50% bone loss).

Evaluation of the distance of the root apex or periapical lesions to the sinus floor

The closest distance of the root apex or periapical lesions to the sinus floor was measured and recorded in the sagittal and coronal views. Direct contact with the sinus was recorded as “0,” whereas the part inside the sinus was recorded as negative.

Since all roots do not have the same relationship with the maxillary sinus, the root/or periapical lesion closest to the sinus floor was recorded.^5^

### Evaluation of maxillary sinuses

**Figure 1:**
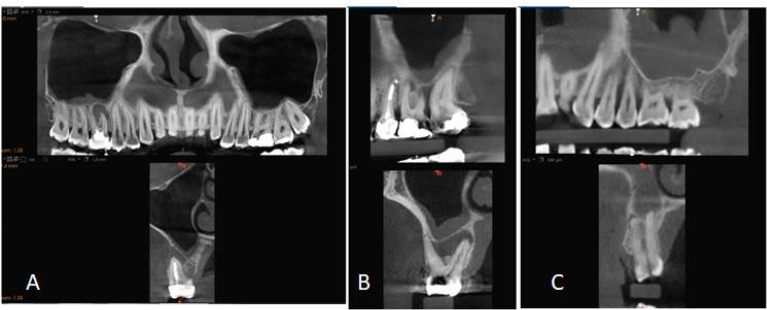
A) Generalized mucosal thickening due to tooth with root canal therapy and apical periodontitis with a well-defined low-density area; B) Severe PBL and periapical lesions and mucosal thickening; C) Polypoid type mucosal thickening and fractured teeth with a periapical lesion.

The maxillary sinuses were imaged in sagittal and coronal views, and the type of MT, MT width, and mucosal appearance (MA) of the maxillary sinuses were evaluated. The type of MT was recorded as follows: normal, local mucosal thickening (> 2 mm, limited to a maximum of two teeth), generalized mucosal thickening (> 2 mm, along the sinus floor), a dome-shaped cyst, or nonspecific opacification or fluid, periostitis, and antrolith (Figure 1).

MT was measured in millimeters, perpendicular to the sinus floor at the highest point of MT on the sagittal view using the software’s measuring tool.^5,13^ The MA was classified as normal, flat thickening, polypoid thickening, or occluded (acute maxillary sinusitis) (Figure 1).

### Statistical analysis

The data were analyzed using SPSS 21 (IBM, Armonk, NY, USA). The Kruskal–Wallis H test was used to compare the groups due to the non-normal distribution of the data. The relationships between categorical variables were analyzed with chi-squared test. Spearman’s correlation analysis was used to determine the relationship between the variables measured. The significance level was determined at *P* < 0.05.

## Results

**Figure 2:**
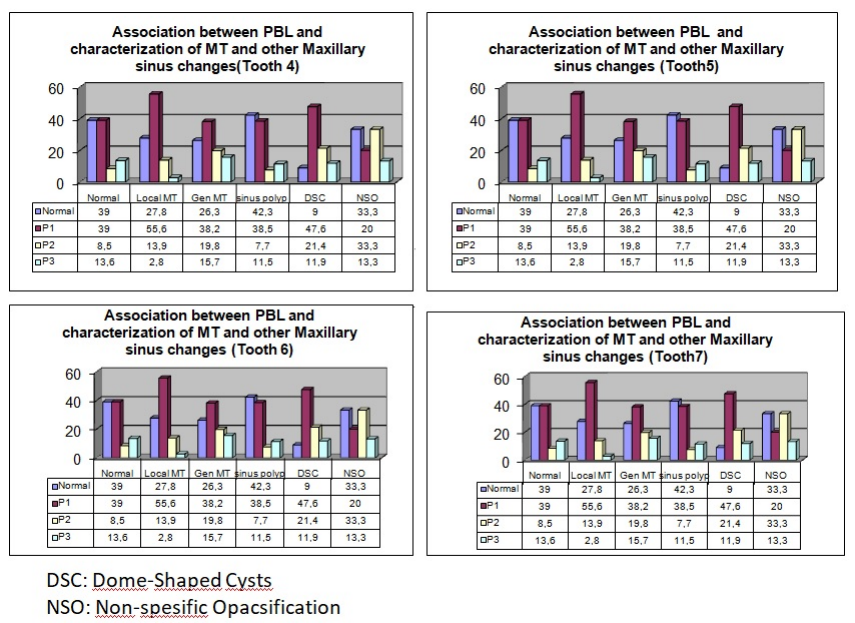
Association between PBL and characterization of MT and other maxillary sinus changes.

**Figure 3:**
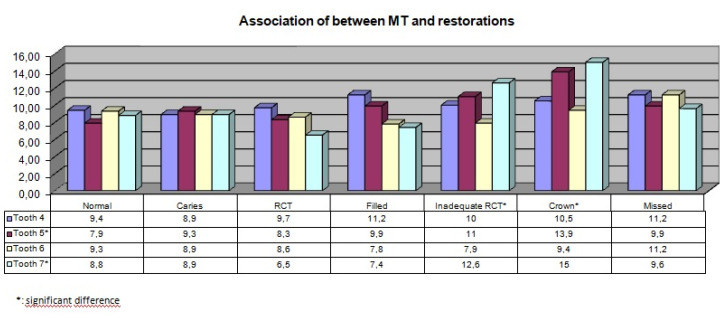
Association between MT and restorations.

**Figure 4:**
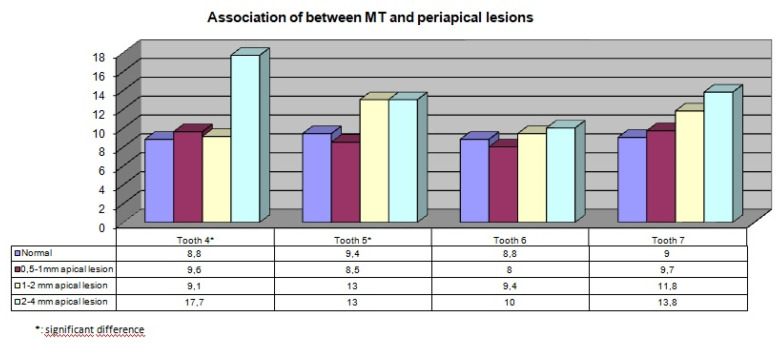
Association between MT and periapical lesions.

Of the 300 patients, 140 (46%) were female, and 160 (54%) were male. There was no statistically significant association between gender and maxillary sinus pathologies (*P* > 0.05). In terms of the association between PBL and the type of MT, a significant relationship was found between a mild degree of PBL in teeth #4 and #6 and generalized MT (*P* = 0.021, *P* = 0.007) (Table 1, Figure 2). Moreover, a significant relationship was found between mild degree of PBL and flat MT increase in tooth #6 (*P* = 0.041). In addition, both localized and generalized MT and dome-shaped cysts were significantly more common in tooth #7 in the presence of a mild degree of PBL (*P* = 0.024, Table 2, Figure 2). Both insufficient root canal treatment and the presence of crown restorations on teeth #5 and #7 were associated with increased MT of maxillary sinuses (*P* < 0.05, Table 3, Figure 3). MT increased significantly in the region of tooth #4 in the presence of periapical lesions measuring 2–4 mm (*P* = 0.022) and in the region of tooth #5 in the presence of periapical lesions measuring 1–2 mm and 2–4 mm (*P* = 0.009, Table 4, Figure 4). No other significant associations were found between the investigated variables and MT.

**Table 1:** Association between PBL and characterization of MT and other Maxillary sinus changes

		**Normal**		**Local MT**		**Genaralize MT**		**Sinus polyp**		**Dome-shaped cyst**		**Non-spesifikopacsifikation or fluid**		**Total**		***P*** ** value**
		**No.**	**%**	**No.**	**%**	**No.**	**%**	**No.**	**%**	**No.**	**%**	**No.**	**%**	**No.**	**%**	
Tooth # 4	Normal	28	49.1	28	39.4	72	28.0	9	36.0	18	47.4	6	40.0	161	34.8	0.021*
	P1	19	33.3	35	49.3	129	50.2	14	56.0	16	42.1	3	20.0	216	46.7	
	P2	6	10.5	7	9.9	35	13.6	0	0.0	3	7.9	4	26.7	55	11.9	
	P3	4	7.0	1	1.4	21	8.2	2	8.0	1	2.6	2	13.3	31	6.7	
	Total	57	100.0	71	100.0	257	100.0	25	100.0	38	100.0	15	100.0	463	100.0	
Tooth # 5	Normal	23	45.1	26	35.6	72	32.0	9	39.1	18	40.9	4	28.6	152	35.3	0.577
	P1	20	39.2	37	50.7	100	44.4	13	56.5	19	43.2	6	42.9	195	45.3	
	P2	6	11.8	6	8.2	36	16.0	0	0.0	4	9.1	2	14.3	54	12.6	
	P3	2	3.9	4	5.5	17	7.6	1	4.3	3	6.8	2	14.3	29	6.7	
	Total	51	100.0	73	100.0	225	100.0	23	100.0	44	100.0	14	100.0	430	100.0	
Tooth # 6	Normal	17	36.2	19	29.2	54	28.1	10	50.0	13	46.4	5	50.0	118	32.6	0.007*
	P1	23	48.9	33	50.8	76	39.6	8	40.0	5	17.9	3	30.0	148	40.9	
	P2	6	12.8	11	16.9	28	14.6	0	0.0	7	25.0	1	10.0	53	14.6	
	P3	1	2.1	2	3.1	34	17.7	2	10.0	3	10.7	1	10.0	43	11.9	
	Total	47	100.0	65	100.0	192	100.0	20	100.0	28	100.0	10	100.0	362	100.0	
Tooth # 7	Normal	23	39.0	20	27.8	57	26.3	11	42.3	8	19.0	5	33.3	124	28.8	0.024*
	P1	23	39.0	40	55.6	83	38.2	10	38.5	20	47.6	3	20.0	179	41.5	
	P2	5	8.5	10	13.9	43	19.8	2	7.7	9	21.4	5	33.3	74	17.2	
	P3	8	13.6	2	2.8	34	15.7	3	11.5	5	11.9	2	13.3	54	12.5	
	Total	59	100.0	72	100.0	217	100.0	26	100.0	42	100.0	15	100.0	431	100.0	

* Significant correlation between characterization of MT and PBL.

**Table 2:** Association of PBL and type of MT

		**MG**										**Chi-square**	***P*** ** value**
		**Normal**		**Flat thickening**		**Polypoid thickening**		**Occluded**		**Total**			
		**No.**	**%**	**No.**	**%**	**No.**	**%**	**No.**	**%**	**No.**	**%**		
Tooth # 4	Normal	30	44.8	61	30.3	60	35.1	11	42.3	162	34.8	16.5	0.056
	P1	25	37.3	99	49.3	87	50.9	6	23.1	217	46.7		
	P2	7	10.4	27	13.4	14	8.2	7	26.9	55	11.8		
	P3	5	7.5	14	7.0	10	5.8	2	7.7	31	6.7		
	Total	67	100.0	201	100.0	171	100.0	26	100.0	465	100.0		
Tooth # 5	Normal	25	41.7	61	32.8	58	36.0	9	37.5	153	35.5	5.6	0.771
	P1	24	40.0	85	45.7	77	47.8	9	37.5	195	45.2		
	P2	8	13.3	28	15.1	14	8.7	4	16.7	54	12.5		
	P3	3	5.0	12	6.5	12	7.5	2	8.3	29	6.7		
	Total	60	100.0	186	100.0	161	100.0	24	100.0	431	100.0		
Tooth # 6	Normal	19	33.3	41	24.8	52	40.6	7	50.0	119	32.7	12.5	0.041*
	P1	28	49.1	72	43.6	46	35.9	3	21.4	149	40.9		
	P2	8	14.0	25	15.2	17	13.3	3	21.4	53	14.6		
	P3	2	3.5	27	16.4	13	10.2	1	7.1	43	11.8		
	Total	57	100.0	165	100.0	128	100.0	14	100.0	364	100.0		
Tooth # 7	Normal	24	36.4	46	25.4	46	28.4	9	39.1	125	28.9	9.04	0.433
	P1	28	42.4	78	43.1	68	42.0	5	21.7	179	41.4		
	P2	8	12.1	32	17.7	30	18.5	4	17.4	74	17.1		
	P3	6	9.1	25	13.8	18	11.1	5	21.7	54	12.5		
	Total	66	100.0	181	100.0	162	100.0	23	100.0	432	100.0		

* Significant correlation between PBL and type of MT.

**Table 3:** Association of between MT and restorations

		**MT**						**Kruskall-Wallis H test**		
		**n**	**Mean**	**Median**	**Minimum**	**Maximum**	**sd**	**Average rank**	**H**	***P*** ** value**
Tooth # 4	Normal	86	9.4	5.8	0.0	48.0	9.7	252.09	7.4	0.283
	Caries	237	8.9	6.1	0.0	41.0	7.9	263.08		
	RCT	15	9.7	6.9	1.5	33.5	8.0	287.90		
	Filled	7	11.2	9.2	3.3	23.0	7.6	327.21		
	Inadequate RCT	39	10.0	7.0	1.2	36.0	8.5	282.12		
	Crown	47	10.5	7.4	1.5	30.1	8.5	293.31		
	Missed	117	11.2	8.3	0.0	43.0	9.3	299.14		
Tooth # 5	Normal	76	7.9	5.3	0.0	37.1	7.5	239.91	13.8	0.031*
	Caries	221	9.3	6.4	0.0	41.0	8.1	268.63		
	RCT	10	8.3	5.7	1.5	28.5	8.4	233.00		
	Filled	5	9.9	4.7	2.9	22.2	8.9	270.00		
	Inadequate RCT	37	11.0	7.3	1.6	38.0	9.9	294.38		
	Crown	45	13.9	12.3	2.1	43.0	10.2	343.66		
	Missed	154	9.9	6.9	0.0	48.0	8.8	277.85		
Tooth # 6	Normal	37	9.3	6.9	0.0	39.0	9.1	268.69	10.1	0.117
	Caries	208	8.9	6.2	0.0	41.0	7.8	259.62		
	RCT	8	8.6	5.5	2.5	22.7	7.5	255.38		
	Filled	14	7.8	5.6	2.6	19.4	5.0	261.39		
	Inadequate RCT	49	7.9	5.5	0.0	36.0	7.1	238.13		
	Crown	19	9.4	6.5	2.5	33.5	8.3	275.11		
	Missed	213	11.2	8.1	0.0	48.0	9.6	299.93		
Tooth # 7	Normal	92	8.8	6.3	0.0	37.1	7.6	264.48	20.3	0.002*
	Caries	206	8.9	6.1	0.0	41.0	8.1	259.14		
	RCT	1	6.5	6.5	6.5	6.5		266.50		
	Filled	16	7.4	6.8	1.7	18.0	4.5	257.50		
	Inadequate RCT	45	12.6	8.5	1.3	38.0	10.3	315.88		
	Crown	36	15.0	13.1	0.0	36.2	9.7	374.86		
	Missed	152	9.6	6.5	1.2	48.0	8.9	267.20		

RCT, Root canal treatment; MT, mucosal thickness. * Significant difference between coronal restorations and MT.

**Table 4:** Association of between MT and periapical lesions

		**MT**						**Kruskall-Wallis H test**		
		**n**	**Mean**	**Median**	**Minimum**	**Maximum**	**sd**	**Average rank**	**H**	***P*** ** value**
Tooth # 4	Normal	202	8.8	5.8	0.0	37.1	8.1	204.14	9.8	0.022*
	0.5-1 mm apical lesion	159	9.6	6.3	0.0	40.0	8.6	216.49		
	1-2 mm apical lesion	55	9.1	7.3	1.2	48.0	8.5	218.96		
	2-4 mm apical lesion	10	17.7	20.1	2.7	30.1	9.2	325.10		
Tooth # 5	Normal	224	9.4	6.7	0.0	41.0	8.3	194.02	11.4	0.009*
	0.5-1 mm apical lesion	110	8.5	6.0	0.0	33.5	7.5	179.62		
	1-2 mm apical lesion	44	13.0	8.4	1.5	43.0	10.7	240.80		
	2-4 mm apical lesion	15	13.0	8.5	2.4	36.0	10.2	240.40		
Tooth # 6	Normal	175	8.8	5.6	0.0	41.0	8.1	163.32	1.14	0.767
	0.5-1 mm apical lesion	80	8.0	6.0	0.0	30.0	6.3	162.14		
	1-2 mm apical lesion	51	9.4	6.8	1.5	36.0	7.9	175.75		
	2-4 mm apical lesion	25	10.0	7.0	0.0	39.0	9.2	177.24		
Tooth # 7	Normal	239	9.0	6.6	0.0	37.1	7.7	187.96	6.2	0.101
	0.5-1 mm apical lesion	70	9.7	6.2	0.0	38.0	8.9	194.34		
	1-2 mm apical lesion	66	11.8	8.2	1.0	41.0	9.8	219.17		
	2-4 mm apical lesion	17	13.8	8.5	2.2	39.0	11.1	237.38		

* Significant difference between periapical lesion and MT.

## Discussion

In this study, mucosal characteristics of maxillary sinusitis, including MT and MA, were evaluated using CBCT images to identify factors (i.e., the coronal, periapical, and periodontal status of the teeth and/or the distance of apical lesions of the maxillary sinus floor) that might account for the etiology of maxillary sinusitis. Since MT was associated with PBL and large periapical lesions, the hypothesis of the current study was partially accepted.

The accurate identification of etiological factors of odontogenic maxillary sinusitis is of utmost importance to ensure selection of the most appropriate treatment and contribute to the prevention of future cases. Although CT is considered the gold standard for imaging paranasal sinuses,^12^ we used CBCT to evaluate maxillary sinuses in the present study due to its low radiation dose, higher resolution quality, and reduced screening time.^5,14^

In the literature on periodontal diseases, apical periodontitis, extracted teeth, and inadequate root canal treatments have been reported to increase maxillary sinusitis-induced MT.^15,16^ According to research, the ideal MT of maxillary sinus ranges from 0.09 mm to 0.97 mm.^16,17^ However, Cagici et al^18^ emphasized that the maxillary sinus MT was only visible when it was > 2 mm. Besides, some studies have advocated that > 2 mm of maxillary sinus MT should be considered pathological.^18-20^ Therefore, the MT of the maxillary sinus > 2 mm was considered pathological in the present study.

Previous studies have shown that periapical lesions close to the sinus floor or associated with the sinus floor increase the likelihood of sinus infections, and larger apical lesions cause an increase in the maxillary sinus MT.^5,21^ Nurbakhsh et al^22^ showed that a decrease in the distance of the tooth apex or lesion from the maxillary sinuses increased the sinus MT. Shanbhag et al^12^ showed that the periapical lesions of molar teeth resulted in increased MT. Goller-Bulut et al^7^ also reported that apical lesions in the region of premolar teeth resulted in increased maxillary sinus MT. However, the same result could not be verified for apical lesions associated with molar teeth. The results of the current study showed that apical lesions measuring 2–4 mm in diameter, associated with premolar teeth, caused increased maxillary sinus MT. However, the same was not true for apical lesions associated with molar teeth, and the current study supported the study by Goller-Bulut et al.^7^

Dental pathology and inadequate root canal treatments are associated with MT in the literature.^7,21^ In the current study, insufficient root canal fillings and crowned teeth were associated with increased sinus MT, consistent with studies by Goller-Bulut et al^7^ and Nenzén and Welander.^23^ In contrast, Phothikhun et al^13^ and Janner et al^24^ found no association between root canal treatments, periapical lesions, and maxillary sinus MT. However, neither Phothikhun et al^13^ nor Janner et al^24^ evaluated the adequacy of root canal treatment, which might have explained the differences between the results.

Periodontitis is an inflammatory disease caused by microorganisms, leading to progressive destruction of the periodontal ligament and alveolar bone.^21^ Previous studies have indicated a direct correlation between PBL and MT of the maxillary sinus in which severe PBL is associated with increased maxillary sinus MT.^7,13,24,25^ Similarly, Shanbhag et al^12^ reported that the PBL caused 2 to 4 mm of MT in the maxillary sinus of their patients. In the current study, the mild degree of MT was significantly associated with teeth #6 and #7, which showed a higher incidence of local MT. The incidence of generalized mucosal thickening was also higher in teeth #4, #6, and #7 with a mild degree of PBL. Presumably, due to the lower incidence of severe PBL, no significant association between severe PBL and maxillary sinus MT was observed in the current study. Moreover, generalized mucosal thickening and dome-shaped cysts were significantly associated with teeth #7 that exhibited a mild degree of PBL.

In contrast to these findings, Yeung et al^26^ observed no association between the periodontal status of the teeth and various mucosal changes in maxillary sinuses. In this study, statistically significant increases were observed in the incidence rate of flat thickening in teeth #6 with mild PBL. To our knowledge, no prior studies have examined the association between PBL and maxillary sinus MT.

Due to its retrospective design, the maxillary sinus MT could not be verified with the patients’ clinical findings, which is a limitation of the current study. Future clinical research is needed to confirm these findings and evaluate the influence of necessary endodontic and/or periodontal treatment on mucosal changes in maxillary sinuses.

## Conclusion

This retrospective study showed a positive correlation between increased maxillary sinus MT and the periapical lesions of premolar teeth and PBL. The polypoid type of MT and dome-shaped cysts were associated with mild degrees of PBL.

## Authors’ Contributions

SIY: concept, design, the definition of intellectual content, literature search, experimental studies, data acquisition, data analysis, statistical analysis, manuscript preparation, manuscript editing, and manuscript review. GNHE and OG: supervision, design, the definition of intellectual content, manuscript editing, and manuscript review.

## Acknowledgments

None.

## Funding

None.

## Competing Interests

The authors declare no conflict of interests.

## Ethics Approval

This study design was approved by the Ethics Committee of Ankara Yıldırım Beyazıt University (No: 2019- 598).
